# Glycemic load, glycemic index, bread and incidence of overweight/obesity in a Mediterranean cohort: the SUN project

**DOI:** 10.1186/1471-2458-14-1091

**Published:** 2014-10-22

**Authors:** Carmen de la Fuente-Arrillaga, Miguel Angel Martinez-Gonzalez, Itziar Zazpe, Zenaida Vazquez-Ruiz, Silvia Benito-Corchon, Maira Bes-Rastrollo

**Affiliations:** Department of Preventive Medicine and Public Health, School of Medicine, University of Navarra, Irunlarrea 1, Pamplona, 31080 Navarra, Spain; CIBER Fisiopatología de la Obesidad y Nutrición (CIBERobn), Instituto de Salud Carlos III (ISCIII), Madrid, Spain; Department of Nutrition and Food Sciences, and Physiology, University of Navarra, Irunlarrea 1, Pamplona, 31080 Navarra, Spain

**Keywords:** Glycemic index, Glycemic load, Bread, Food-frequency questionnaire, SUN (Seguimiento Universidad de Navarra) project

## Abstract

**Background:**

To evaluate prospectively the relationship between white, or whole grain bread, and glycemic index, or glycemic load from diet and weight change in a Mediterranean cohort.

**Methods:**

We followed-up 9 267 Spanish university graduates for a mean period of 5 years. Dietary habits at baseline were assessed using a semi-quantitative 136-item food-frequency questionnaire. Average yearly weight change was evaluated according to quintiles of baseline glycemic index, glycemic load, and categories of bread consumption. We also assessed the association between bread consumption, glycemic index, or glycemic load, and the incidence of overweight/obesity.

**Results:**

White bread and whole-grain bread were not associated with higher weight gain. No association between glycemic index, glycemic load and weight change was found.

White bread consumption was directly associated with a higher risk of becoming overweight/obese (adjusted OR (≥2 portions/day) versus (≤1 portion/week): 1.40; 95% CI: 1.08-1.81; p for trend: 0.008). However, no statistically significant association was observed between whole-grain bread, glycemic index or glycemic load and overweight/obesity.

**Conclusions:**

Consumption of white bread (≥2 portions/day) showed a significant direct association with the risk of becoming overweight/obese.

## Background

Worldwide, in the last two decades, the prevalence of obesity and obesity-related chronic diseases has increased [1]. Therefore, the identification of simple, cost-effective strategies for the prevention and management of obesity is urgently needed [2].

Habitual diet together with sedentary lifestyles are the major modifiable factors determining body weight gain [3]. Thus, it is hypothesized that habitual consumption of carbohydrate-rich foods may promote the risk of developing obesity [4]. However the role of carbohydrates in the prevention and management of obesity is not completely clear and the results are inconsistent [2].

Carbohydrates are the main component of the diet and are typically categorized into simple sugars and complex carbohydrates on the basis of their chemical structure. However, their effects on health may be better categorized according to insulin secretion and postprandial glycemia [5].

On one hand, the concept of Glycemic Index (GI), developed in the early 1980s by Jenkins et al. [6], is a quantitative measure of carbohydrate quality based on the blood glucose response after consumption. On the other hand, the concept of Glycemic Load (GL), defined later, has been proposed as a global indicator of the glucose response and insulin demand induced by a serving of food [7]. GL is calculated as the mathematical product of the GI of a food multiplied by its carbohydrate content.

Few cross-sectional studies and only four longitudinal studies have assessed the relationship between GI or GL and body weight or weight changes [3, 8–10].

Their results are not fully consistent [10]. Furthermore, to our knowledge, only two prospective studies have been conducted in a Mediterranean population assessing the effect of bread consumption as a risk factor for obesity: the EPIC cohort [11] and a subsample of the PREDIMED trial [12]. Consequently, the purpose of our prospective analysis was to examine the association between dietary GI, GL or bread consumption and the average weight gain during follow-up (or the risk of becoming overweight/obese) in a large prospective Mediterranean cohort of university graduates.

## Methods

### Study population

The objectives, design, and methods of the SUN (“Seguimiento Universidad de Navarra”: University of Navarra follow-up) project have been described elsewhere [13]. The SUN project is a multipurpose, dynamic cohort designed to assess the association between diet and several chronic diseases and health conditions. The recruitment of participants started in December 1999, and additional questionnaires are mailed every 2 years.

Participants who completed a baseline assessment (Q_0) before February 2006, and therefore were able to provide at least their 2-year follow-up information were eligible for these longitudinal analyses (n = 15 982).

Among them, 1 885 had not answered any of the follow-up questionnaires, and after five more mailings separated by 3 months each, they were considered lost to follow-up. Therefore, we retained 14 097 (88%) of the candidate participants. Among them, participants who had some of the following characteristics were excluded from the analyses: pregnant women at baseline or during follow-up (n = 1 272), those with missing data in variables of interest (n = 14), or with extreme values for total energy intake (<800 or >4 000 kcal/day for men and <500 or >3 500 kcal/day for women) (n = 1 380) [14]. We also excluded those who were following a special diet at baseline (n = 922), and those participants with chronic disease (cardiovascular disease, diabetes or cancer) at baseline or during follow-up (n = 1 242). Finally, data from 9 267 participants remained available for the analyses.

The Institutional Review Board at the University of Navarra approved the study protocol. We considered a response to the initial questionnaire as informed consent to participate in the study.

### Assessment of dietary exposure

Dietary habits at baseline were assessed using a Food-Frequency Questionnaire (FFQ) with 136 items, previously validated in Spain [15, 16]. This questionnaire assessed food habits in the previous year. There were 9 possible answers (ranging from never/almost never to 6+ times per day). The questionnaire was semi-quantitative, i.e., for each food, a standard portion size was specified. Nutrient intake was calculated by multiplying the frequency of consumption by the nutrient content of the specified portion, using data from Spanish food composition tables [17].

For the purpose of this study, the GI for food and beverage items was estimated by using average values from the 2002 International tables of GI and GL values and expanded in 2008 [18] with glucose as the reference food.

Dietary GL was calculated taking into account the quality and the amount of carbohydrate [GL = (GI x amount of available carbohydrate)/100] [19]. Finally, both dietary GI and dietary GL were categorized into quintiles.

Bread consumption was assessed through two specific questions of the FFQ based on the daily consumption of white bread or whole-grain bread in the previous year. One portion is specified in the FFQ as 60 g or 3 slices. Participants were categorized in 4 groups: ≤1/week, 2-6/week, 1/day and ≥2/day.

Adherence to the traditional Mediterranean diet was assessed by a 10-point Mediterranean-diet scale that incorporated the salient characteristics of this diet [20].

### Assessment of other variables

The baseline questionnaire also collected information on a wide array of characteristics, including sociodemographic variables, health-related habits, and clinical variables.

We assessed physical activity at baseline using a previously validated questionnaire which included information about 17 activities [21]. The time spent in different activities was multiplied by the MET (Metabolic Equivalent Score) specific to each activity [22], and then the MET score were summed over all activities to obtain a value of overall weekly MET hours.

### Assessment of the outcome

Information on weight was collected at baseline and at each follow-up questionnaire. 1 426 participants were followed-up for 8 years, 3 008 for 6 years, 2 567 for 4 years, and 2 266 for 2 years (mean period of follow-up 5 years). The reproducibility and validity of self-reported weight were assessed in a subsample of the cohort [23].

The outcomes were: 1) average yearly change in body weight (g/year) during follow-up as a continuous variable [(weight in the last answered questionnaire – weight in the baseline questionnaire) / years of follow-up] and 2) incident of overweight or obesity (BMI <25 kg/m^2^ at baseline and with a BMI ≥25 kg/m^2^ in any point during follow-up).

### Statistical analysis

Multivariable linear regression models were used to assess the association between baseline dietary GI or dietary GL and average weight change per year. Non-conditional logistic regression models were fit to assess the relationship between baseline dietary GI or dietary GL (both categorized in quintiles), categories of bread consumption (4 categories), and the risk of incident overweight/obesity (BMI ≥25 kg/m^2^) during the follow-up period for participants with BMI <25 kg/m^2^ at baseline.

Tests of linear trend across increasing categories or quintiles of dietary exposures were calculated for the models assessing weight change or the risk of overweight/obesity. To analyse these trends the median value of GI, GL, or bread consumption was imputed for each category or quintile and we considered the new variable as a continuous one.

For each exposure, we fitted 5 types of models: a) an age- and sex- adjusted model; b) a multivariate- adjusted model controlling for age, sex, baseline BMI (kg/m^2^, continuous), smoking status (never smoker, ex-smoker and current smoker), physical activity during leisure time (MET-hours/week, continuous), total time of sedentary activities (h/week, continuous) and time spent in TV watching (h/week, continuous); c) a multivariate- adjusted model, adjusted for fiber intake and total energy intake in addition to all the variables mentioned above; d) we additionally adjusted also for protein intake; e) finally, we adjusted for all the variables mentioned above but replacing protein intake for olive oil intake.

In all analyses, the lowest quintile of dietary GI or GL or the lowest category of bread consumption (≤1 portion/week) were considered as the reference category.

To evaluate the main source of variability in GI and GL we used the cumulative R^2^ values in stepwise regression analysis [24].

All *P* values are two-tailed; *P* <0.05 was considered statistically significant. Analyses were performed using SPSS version 15.0 (SPSS Inc. Chicago, IL, USA).

## Results and discussion

The mean age at baseline was 38 years (54% women) and participants were followed for a mean period of 5 years.

The baseline characteristics of the participants across quintiles of dietary GI are presented in Table 1. The mean dietary GI was 52 (SD: 4). Women were more likely than men to be in the lowest quintile. Higher intakes of total energy, whole grain bread, soft drinks and olive oil were associated with a higher dietary GI. Participants with a higher intake of protein, total fat, saturated fat and monounsaturated fat reported lower dietary GI.

**Table 1:** **Main characteristics (mean and standard deviation (s.d.)) of the 9 267 participants of the SUN project according to quintiles of glycemic index and glycemic load**

Glycemic index	Quintile 1	Quintile 2	Quintile 3	Quintile 4	Quintile 5	***P*** ^***a***^
Participants (n)	1 859	1 851	1 852	1 853	1 852	
Glycemic index	45 (2)	50 (0.7)	52 (0.6)	54 (0.7)	58 (2)	<0.001
Age (years)	39.1 (11.5)	37.3 (11.1)	36.9 (11.3)	37.3 (11.3)	38.0 (11.2)	<0.001
Baseline BMI (kg/m^2^)	23.7 (3.3)	23.5 (3.3)	23.4 (3.2)	23.4 (3.2)	23.6 (3.3)	0.032
Baseline weight (kg)	67.4 (13.5)	67.6 (13.4)	67.3 (13.1)	67.6 (13.1)	69.0 (13.2)	<0.001
Physical activity during leisure time (MET-h/week)	25.0 (24.1)	24.8 (22.3)	24.4 (22.0)	25.3 (21.6)	22.8 (20.4)	<0.001
Weight change (kg/year)	0.2 (1.0)	0.2 (1.0)	0.2 (1.0)	0.1 (0.9)	0.2 (1.0)	0.17
TV (h/day)	1.6 (1.2)	1.6 (1.2)	1.6 (1.2)	1.6 (1.1)	1.6 (1.2)	0.54
Sitting (h/day)	2.9 (2.3)	3.0 (2.4)	2.9 (2.3)	2.9 (2.4)	3.1 (2.4)	0.24
Sex (%)						<0.001
Men	39.4	43.3	44.1	47.5	55.0	
Smoking status (%)						0.003
Current smoker	26.8	25.7	25.5	25.6	25.4	
Ex-smoker	30.5	28.4	25.6	27.9	27.1	
Energy (kcal/day)	2 130 (608)	2 335 (594)	2 413 (595)	2 512 (601)	2 576 (594)	<0.001
Carbohydrates (% E)	39 (7)	42 (6)	43 (6)	44 (6)	47 (6)	<0.001
Protein (% E)	20 (3)	18 (2)	17 (2)	16 (2)	16 (2)	<0.001
Fat (% E)	38 (7)	37 (6)	37 (5)	36 (5)	33 (6)	<0.001
SFA (% E)	13.4 (3.9)	12.9 (3.1)	12.9 (2.9)	12.4 (2.8)	11.5 (2.7)	<0.001
MUFA (% E)	16.3 (4.1)	15.8 (3.5)	15.8 (3.4)	15.6 (3.4)	14.7 (3.5)	<0.001
PUFA (% E)	5.0 (1.5)	5.3 (1.5)	5.4 (1.6)	5.4 (1.6)	5.2 (1.6)	<0.001
Fiber (g/day)	27.8 (14.0)	27.2 (11.6)	26.5 (11.3)	26.3 (10.6)	25.2 (10.7)	<0.001
Pure alcohol (g/day)	8.6 (13.6)	6.8 (10.4)	6.5 (9.4)	6.6 (9.0)	6.6 (10.0)	0.84
Vegetables (g/day)	637 (425)	533 (298)	475 (269)	442 (245)	383 (219)	<0.001
Fruit (g/day)	373 (314)	364 (313)	339 (293)	312 (256)	251 (208)	<0.001
Legumes (g/day)	23 (19)	24 (17)	23 (18)	22 (16)	20 (12)	<0.001
White bread (g/day)	18 (23)	35 (28)	49 (36)	78 (54)	143 (90)	<0.001
Whole grain bread (g/day)	6 (16)	9 (21)	11 (26)	11 (28)	16 (46)	<0.001
Dairy products (g/day)	212 (235)	227 (211)	230 (203)	222 (201)	208 (181)	0.001
Meat and meat products (g/day)	174 (84)	179 (76)	179 (72)	177 (73)	173 (72)	0.028
Fish and seafood (g/day)	106 (67)	102 (65)	93 (54)	91 (52)	84 (48)	<0.001
Processed pastries (g/day)	11 (16)	15 (22)	16 (22)	16 (21)	15 (22)	<0.001
Soft-drinks (g/day)	55 (116)	63 (130)	61 (99)	65 (121)	66 (138)	0.044
Fast-food (g/day)	19 (21)	22 (21)	22 (19)	21 (19)	19 (18)	<0.001
Olive oil (g/day)	19 (17)	19 (16)	19 (16)	21 (17)	22 (19)	<0.001
Mediterranean dietary pattern^b^	4.2 (1.7)	4.1 (1.8)	4.1 (1.8)	4.3 (1.8)	4.2 (1.7)	0.017
**Glycemic load**	**Quintile1**	**Quintile 2**	**Quintile 3**	**Quintile 4**	**Quintile 5**	
Participants (n)	1 851	1 858	1 853	1 850	1 855	
Glycemic load	73 (17)	109 (7)	134 (7)	161 (8)	213 (31)	<0.001
Age (years)	39.4 (11.5)	37.4 (11.2)	37.2 (11.1)	36.8 (11.3)	37.8 (11.5)	<0.001
Baseline BMI (kg/m^2^)	24.0 (3.5)	23.5 (3.2)	23.4 (3.2)	23.2 (3.2)	23.5 (3.2)	<0.001
Baseline weight (kg)	68.3 (13.8)	67.5 (13.3)	67.0 (12.8)	67.0 (13.4)	69.1 (13.0)	<0.001
Physical activity during leisure time (MET-h/week)	21.3 (18.8)	23.4 (19.9)	24.5 (22.5)	25.8 (23.0)	27.3 (25.4)	<0.001
Weight change (kg/year)	0.2 (1.0)	0.2 (0.9)	0.2 (0.9)	0.2 (1.0)	0.1 (0.9)	0.31
TV (h/day)	1.6 (1.1)	1.6 (1.2)	1.6 (1.2)	1.6 (1.2)	1.6 (1.2)	0.13
Sitting (h/day)	2.9 (2.3)	3.0 (2.3)	2.9 (2.4)	3.0 (2.4)	3.1 (2.4)	0.05
Sex (%)						<0.001
Men	42.9	42.4	42.8	45.6	55.6	
Smoking status (%)						<0.001
Current smoker	27.9	26.7	25.6	25.8	23.2	
Ex-smoker	31.2	29.0	28.3	26.2	24.7	
Energy (kcal/day)	1 664 (390)	2 112 (349)	2 402 (378)	2 686 (373)	3 102 (395)	<0.001
Carbohydrates (% E)	37 (7)	41 (5)	43 (5)	45 (5)	49 (5)	<0.001
Protein (% E)	20 (3)	18 (2)	17 (2)	17 (2)	15 (2)	<0.001
Fat (% E)	39 (7)	37 (6)	36 (5)	35 (5)	32 (5)	<0.001
SFA (% E)	13.9 (3.7)	13.0 (3.1)	12.7 (2.9)	12.2 (2.7)	11.1 (2.6)	<0.001
MUFA (% E)	17.2 (4.4)	16.1 (3.5)	15.6 (3.3)	15.2 (3.1)	13.9 (2.9)	<0.001
PUFA (% E)	5.3 (1.6)	5.3 (1.6)	5.3 (1.6)	5.3 (1.6)	5.0 (1.5)	<0.001
Fiber (g/day)	18 (8)	23 (9)	25 (8)	29 (10)	34 (13)	<0.001
Pure alcohol (g/day)	7.2 (11.4)	7.1 (10.2)	6.9 (10.2)	6.8 (10.3)	7.1 (11.1)	0.84
Vegetables (g/day)	428 (284)	489 (315)	491 (285)	523 (321)	538 (340)	<0.001
Fruit (g/day)	212 (163)	288 (211)	324 (234)	362 (268)	451 (416)	<0.001
Legumes (g/day)	17 (13)	21 (14)	23 (15)	24 (18)	26 (21)	<0.001
White bread (g/day)	21 (24)	39 (34)	57 (46)	78 (57)	128 (97)	<0.001
Whole grain bread (g/day)	5 (14)	8 (20)	10 (25)	13 (34)	17 (43)	<0.001
Meat and meat products (g/day)	154 (75)	171 (75)	184 (75)	188 (73)	185 (74)	<0.001
Fish and seafood (g/day)	88 (65)	95 (55)	97 (54)	96 (53)	101 (62)	<0.001
Processed pastries (g/day)	8 (12)	12 (16)	15 (20)	17 (22)	20 (28)	<0.001
Soft-drinks (g/day)	51 (112)	53 (87)	64 (120)	62 (127)	79 (150)	<0.001
Fast-food (g/day)	15 (16)	20 (17)	22 (20)	24 (21)	23 (21)	<0.001
Olive oil (g/day)	16 (16)	19 (17)	20 (16)	22 (18)	22 (18)	<0.001
Mediterranean dietary pattern^b^	3.5 (1.6)	3.9 (1.7)	4.2 (1.8)	4.5 (1.7)	4.8 (1.7)	<0.001

^a^P value for comparison between-groups calculated by one-factor ANOVA for continuous variables or the *χ* ^2^ test for categorical variables.

^b^Trichopoulou score (range of scores, 0 to 9, with higher scores indicating greater adherence).

Table 1 shows also the characteristics of study participants across quintiles of GL. The mean dietary GL was 138 (SD: 29). A high dietary GL was observed among men, among participants who were more active during leisure time and among never smokers. Energy from carbohydrates and dietary fiber intakes increased in parallel with GL. In addition, participants in the higher quintile of GL had also higher consumption of vegetables, fruits, legumes, whole grain bread, dairy products, pastries and olive oil.

In relation to the Mediterranean dietary pattern, significant differences were observed across quintiles of GI and of GL.

The main characteristics of the participants according to categories of white bread and whole-grain bread are presented in Table 2. Higher white bread consumption was observed among men, older people, among participants with a higher BMI, higher energy intake, higher percentage of carbohydrates and lower of protein and fat, higher fiber, alcohol, dairy products, meat and meat products, processed pastries, and olive oil intake. No differences were observed for physical activity, sedentary habits or smoking status.

**Table 2:** **Main characteristics (mean and standard deviation (s.d.)) of the 9 267 participants of the SUN project according to categories of white bread and whole-grain bread consumption** ^**a**^

White bread	≤ 1/week	2-6/week	1/day	≥ 2/day	***P*** ^***b***^
Participants (n)	2 474	2 010	2 680	2 103	
White bread (g/day)	3 (4)	36 (11)	60 (0)	171 (62)	<0.001
Age (years)	37.7 (11.7)	37.2 (11.3)	37.0 (10.9)	39.2 (11.6)	<0.001
Baseline BMI (kg/m^2^)	23.5 (3.4)	23.6 (3.3)	23.3 (3.2)	23.9 (3.4)	<0.001
Baseline weight (kg)	66.8 (13.4)	68.1 (13.5)	66.8 (12.8)	70.2 (13.4)	<0.001
Physical activity during leisure time (MET-h/week)	25.1 (23.1)	24.3 22.8	24.3 21.8	24.3 20.9	0.45
Weight change (kg/year)	0.2 (1)	0.3 (1)	0.2 (0.9)	0.3 (1)	0.14
TV (h/day)	1.7 (1.3)	1.6 (1.2)	1.6 (1.3)	1.6 (1.2)	0.78
Sitting (h/day)	2.9 (2.4)	3.1 (2.4)	3.0 (2.5)	3.1 (2.5)	0.09
Sex (%)					<0.001
Men	38.4	46.8	41.6	59.2	
Smoking status (%)					0.32
Current smoker	26.8	26.8	24.6	25.2	
Ex-smoker	27.4	26.7	28.1	29.1	
Energy (kcal/day)	2 133 (629)	2 261 (570)	2 441 (552)	2 767 (532)	<0.001
Carbohydrates (% E)	41 (8)	43 (6)	44 (6)	47 (6)	<0.001
Protein (% E)	19 (4)	18 (3)	18 (3)	17 (2)	<0.001
Fat (% E)	38 (7)	38 (6)	37 (6)	34 (6)	<0.001
SFA (% E)	13.2 (3.8)	13.0 (2.9)	12.7 (2.8)	11.6 (2.5)	<0.001
MUFA (% E)	16.2 (4.3)	15.6 (3.2)	15.8 (3.4)	14.7 (3.4)	<0.001
PUFA (% E)	5.3 (1.7)	5.4 (1.5)	5.3 (1.5)	5.0 (1.5)	<0.001
Fiber (g/day)	27 14)	25 (11)	27 (11)	28 (10)	<0.001
Pure alcohol (g/day)	6.5 (11.1)	6.9 (9.9)	6.7 (9.8)	8.3 (11.6)	<0.001
Vegetables (g/day)	525 (364)	468 (283)	504 (297)	473 (289)	<0.001
Fruit (g/day)	343 (313)	298 (233)	354 (311)	307 (249)	<0.001
Legumes (g/day)	24 (25)	23 (14)	22 (12)	23 (14)	<0.001
Whole grain bread (g/day)	21 (41)	9 (23)	9 (26)	6 (22)	<0.001
Dairy products (g/day)	196 (211)	208 (193)	237 (210)	240 (209)	<0.001
Meat and meat products (g/day)	167 (84)	178 (75)	179 (72)	185 (71)	<0.001
Fish and seafood (g/day)	98 (66)	97 (57)	96 (59)	92 (50)	0.001
Processed pastries (g/day)	12 (19)	15 (20)	16 (22)	17 (24)	<0.001
Soft-drinks (g/day)	67 (150)	66 (118)	59 (95)	58 (119)	0.033
Fast-food (g/day)	19 (20)	23 (21)	22 (20)	21 (19)	<0.001
Olive oil (g/day)	19 (18)	16 (14)	22 (17)	25 (20)	<0.001
Mediterranean dietary pattern^b^	4.0 (1.8)	3.9 (1.8)	4.3 (1.8)	4.7 (1.7)	<0.001
**Whole-grain bread**	**≤ 1/week**	**2-6/week**	**1/day**	**≥ 2/day**	***P*** ^***b***^
Participants (n)	7672	771	603	221	
Whole grain bread (g/day)	1 (2)	32 (10)	60 (0)	162 (47)	<0.001
Age (years)	37.7 (11.4)	37.6 (11.1)	37.9 (11.6)	41.1 (11.6)	<0.001
Baseline BMI (kg/m2)	23.6 (3.3)	23.5 (3.4)	23.2 (3.2)	23.3 (3.1)	0.006
Baseline weight (kg)	68.2 (13.4)	67.1 (13.7)	64.8 (11.9)	65.6 (12.3)	<0.001
Physical activity during leisure time (MET-h/week)	23.9 (21.5)	27.4 (26.2)	25.8 (22.4)	30.3 (25.6)	<0.001
Weight change (kg/year)	0.23 (0.9)	0.26 (1.1)	0.23 (1)	0.09 (0.82)	0.16
TV (h/day)	1.6 (1.2)	1.6 (1.1)	1.7 (1.4)	1.7 (1.4)	0.21
Sitting (h/day)	3.0 (2.4)	2.9 (2.3)	2.9 (2.6)	2.8 (2.2)	0.06
Sex (%)					<0.001
Men	48.4	36.6	29.0	34.8	
Smoking status (%)					0.14
Current smoker	26.4	23.3	23.2	19.5	
Ex-smoker	27.3	29.1	31.0	33.9	
Energy (kcal/day)	2384 (625)	2323 (580)	2478 (572)	2733 (513)	<0.001
Carbohydrates (% E)	44 (7)	44 (7)	45 (7)	49 (7)	<0.001
Protein (% E)	18 (3)	18 (3)	18 (3)	17 (3)	<0.001
Fat (% E)	37 (6)	35 (6)	35 (7)	33 (6)	<0.001
SFA (% E)	12.9 (3.1)	11.9 (3.0)	11.3 (2.9)	10.2 (2.5)	<0.001
MUFA (% E)	15.7 (3.6)	14.9 (3.4)	15.1 (3.7)	14.4 (3.8)	<0.001
PUFA (% E)	5.3 (1.6)	4.9 (1.3)	4.9 (1.5)	4.6 (1.3)	<0.001
Fiber (g/day)	25 (11)	30 (12)	35 (12)	44 (13)	<0.001
Pure alcohol (g/day)	7.2 (10.9)	6.4 (8.4)	5.9 (9.5)	6.2 (9.8)	0.008
Vegetables (g/day)	475 (306)	575 (323)	606 (329)	588 (315)	<0.001
Fruit (g/day)	313 (276)	365 (280)	427 (317)	454 (362)	<0.001
Legumes (g/day)	23 (18)	23 (15)	23 (15)	19 (9)	0.016
White bread (g/day)	70 (70)	36 (47)	43 (52)	33 (53)	<0.001
Dairy products (g/day)	230 (212)	179 (185)	170 (166)	164 (169)	<0.001
Meat and meat products (g/day)	180 (76)	154 (76)	164 (81)	156 (71)	<0.001
Fish and seafood (g/day)	94 (59)	105 (56)	105 (59)	109 (62)	<0.001
Processed pastries (g/day)	16 (22)	12 (16)	10 (14)	11 (19)	<0.001
Soft-drinks (g/day)	64 (123)	63 (126)	51 (101)	42 (118)	0.006
Fast-food (g/day)	22 (20)	21 (21)	18 (17)	15 (15)	<0.001
Olive oil (g/day)	20 (18)	19 (15)	25 (19)	29 (20)	<0.001
Mediterranean dietary pattern^c^	4.0 (1.7)	4.8 (1.7)	5.2 (1.7)	5.5 (1.6)	<0.001

^a^One portion of white bread or whole-grain bread was specified as 60 g or 3 slices.

^b^P value for comparison between-groups calculated by one-factor ANOVA for continuous variables or the *χ* ^2^ test for categorical variables.

^c^Trichopoulou score (range of scores, 0 to 9, with higher scores indicating greater adherence).

Participants in the highest whole-grain bread consumption category, were more like to be older, women, more physically active, and had a lower baseline weight. Moreover, they had a higher total energy intake and the highest intake of fiber and fruits and vegetables consumption.

Referring to the Mediterranean dietary pattern, significant differences (*P* <0.001) were observed across categories of white bread and of whole-grain bread consumption.

The inter-individual variation, in both dietary GI and GL was explained in first place by white bread. White bread explained 42% of the variability in GI and 35% in GL. 51% of the variability in GL was explained by white bread, fried potatoes, and whole grain bread.

The results of the multivariable linear regression models fitted to evaluate the association between baseline dietary GI or GL and yearly weight gain during follow-up, showed that although some point estimates suggested an inverse association between GI and weight gain, none of the adjusted-models found a significant association (*P* for trend = 0.12). In contrast, after adjustment for potential confounding variables (age, sex, physical activity, total time of sedentary activities, smoking status, baseline BMI, time spent in TV watching, fiber intake, energy intake, and olive oil consumption), GL was inversely associated with average yearly weight change. Thus, we found a slightly lower average body weight gain (g per year) among participants in the fifth quintile (ß = −148; 95% CI: −252 to −44) compared with those in the lowest quintile after adjusting for potential confounders (P for trend = 0.002). However, when we repeated the analyses adjusting also for protein percentage, the results did not remain statistically significant (data not shown).

To examine the association between GI or GL and the risk of becoming overweight/obese, we included 6 496 subjects without prevalent overweight or obesity at baseline. After follow-up, we observed 943 new cases of overweight/obesity.

No trends were observed across quintiles of dietary GI for the risk of overweight/obesity (Table 3).

**Table 3:** **Odds ratios and 95% CI of incident overweight or obesity at follow-up in 6 496 participants of the SUN project according to quintiles of glycemic index and glycemic load**

			Quintiles	Glycemic	Index	
	Q1	Q2	Q3	Q4	Q5	p for trend
Participants (n)	1 270	1 304	1 324	1 316	1 282	
Incident cases overweight/obesity	178	189	188	177	211	
Age- and sex-adjusted OR (95% CI)	1 (Ref.)	0.98 (0.78-1.22)	0.93 (0.74-1.17)	0.82 (0.65-1.03)	0.95 (0.76-1.19)	0.342
Multivariate adjusted OR^1^ (95% CI)	1 (Ref.)	1.02 (0.79-1.32)	0.99 (0.76-1.29)	0.83 (0.64-1.08)	1.12 (0.87-1.45)	0.807
Multivariate adjusted OR^2^ (95% CI)	1 (Ref.)	1.00 (0.77-1.30)	0.97 (0.74-1.26)	0.80 (0.61-1.05)	1.07 (0.82-1.40)	0.907
Multivariate adjusted OR^3^ (95% CI)	1 (Ref.)	0.99 (0.76-1.30)	0.96 (0.73-1.26)	0.79 (0.60-1.05)	1.06 (0.80-1.40)	0.871
Multivariate adjusted OR^4^ (95% CI)	1 (Ref.)	1.00 (0.77-1.30)	0.97 (0.74-1.26)	0.80 (0.61-1.05)	1.07 (0.80-1.40)	0.785
			**Quintiles**	**Glycemic**	**Load**	
	**Q1**	**Q2**	**Q3**	**Q4**	**Q5**	**p for trend**
Participants (n)	1 186	1 321	1 318	1 368	1 303	
Incident cases overweight/obesity	166	219	187	182	189	
Age- and sex-adjusted OR (95% CI)	1 (Ref.)	1.19 (0.95-1.49)	0.98 (0.78-1.24)	0.86 (0.68-1.08)	0.81 (0.64-1.03)	0.004
Multivariate adjusted OR^1^ (95% CI)	1 (Ref.)	1.21 (0.93-1.57)	1.04 (0.80-1.36)	0.96 (0.74-1.25)	1.02 (0.78-1.33)	0.516
Multivariate adjusted OR^2^ (95% CI)	1 (Ref.)	1.12 (0.85-1.47)	0.91 (0.67-1.24)	0.79 (0.56-1.12)	0.77 (0.51-1.18)	0.075
Multivariate adjusted OR^3^ (95% CI)	1 (Ref.)	1.09 (0.83-1.45)	0.88 (0.64-1.22)	0.76 (0.53-1.10)	0.73 (0.47-1.15)	0.053
Multivariate adjusted OR^4^ (95% CI)	1 (Ref.)	1.12 (0.85-1.48)	0.92 (0.67-1.30)	0.80 (0.56-1.14)	0.78 (0.51-1.20)	0.064

Q1-Q5: lowest to highest quintile.

*OR* Odd Ratio.

*CI* Confidence Interval.

^1^adjusted by age, sex, physical activity, time spent in TV watching, total time of sedentary activities, smoking status, baseline BMI.

^2^adjusted by age, sex, physical activity, time spent in TV watching, total time of sedentary activities, smoking status, baseline BMI, fiber intake, and total energy intake.

^3^adjusted by age, sex, physical activity, time spent in TV watching, total time of sedentary activities, smoking status, baseline BMI, fiber intake, total energy intake, and protein percentage.

^4^adjusted by age, sex, physical activity, time spent in TV watching, total time of sedentary activities, smoking status, baseline BMI, fiber intake, total energy intake, and olive oil consumption.

Participants in the fifth quintile of dietary GL had an apparent reduced risk of becoming overweight/obese (OR = 0.81; 95% CI: 0.64 to 1.03) after adjusting for age and sex (*P* for trend = 0.004). However, when we repeated the analyses adjusting for other potential confounding variables, the association remained only marginally significant (*P* for trend = 0.064) (Table 3).

We evaluated the association among baseline consumption of white bread, or whole-grain bread, and the average early weight gain during follow-up. After adjustment for potential confounding variables, categories of consumption of white bread or whole-grain bread were not associated with average yearly weight gain (data not shown).

Participants in the highest category of white bread consumption (≥2 portions/day, ≥6 slices/day) showed a significantly increased risk of becoming overweight/obese when we adjusted for all potential confounding variables compared to those participants with the lowest consumption (≤1 portion/week, ≤3 slices/week) (OR: 1.40; 95% CI: 1.08 to 1.81; P for trend = 0.008) (Table 4).

**Table 4:** **Odds ratios and 95% CI of incident overweight or obesity at follow-up in 6 496 participants of the SUN project according to categories of white bread and whole-grain bread consumption** ^**a**^

White bread		Frequency	Consumption	Categories	
	≤ 1/week	2-6/week	1/day	≥ 2/day	p for trend
Participants (n)	1 755	1 411	1 939	1 391	
Incident cases overweight/obesity	214	211	261	257	
Age- and sex-adjusted OR (95% CI)	1 (Ref.)	1.13 (0.91-1.39)	1.06 (0.87-1.30)	1.23 (1.00-1.51)	0.066
Multivariate adjusted OR^1^ (95% CI)	1 (Ref.)	1.14 (0.89-1.45)	1.10 (0.88-1.38)	1.39 (1.10-1.76)	0.006
Multivariate adjusted OR^2^ (95% CI)	1 (Ref.)	1.13 (0.89-1.44)	1.10 (0.87-1.39)	1.40 (1.08-1.80)	0.011
Multivariate adjusted OR^3^ (95% CI)	1 (Ref.)	1.13 (0.89-1.44)	1.11 (0.88-1.40)	1.40 (1.08-1.82)	0.011
Multivariate adjusted OR^4^ (95% CI)	1 (Ref.)	1.14 (0.90-1.46)	1.11 (0.88-1.40)	1.40 (1.08-1.81)	0.008
Multivariate adjusted OR^5^ (95% CI)	1 (Ref.)	1.14 (0.90-1.50)	1.12 (0.89-1.41)	1.43 (1.11-1.86)	0.015
**Whole-grain bread**		**Frequency**	**Consumption**	**Categories**	
	**≤ 1/week**	**2-6/week**	**1/day**	**≥ 2/day**	**p for trend**
Participants (n)	5 336	543	456	161	
Incident cases overweight/obesity	804	72	52	15	
Age- and sex-adjusted OR (95% CI)	1 (Ref.)	1.01 (0.78-1.33)	0.87 (0.64-1.19)	0.63 (0.36-1.10)	0.089
Multivariate adjusted OR^1^ (95% CI)	1 (Ref.)	1.06 (0.78-1.44)	0.83 (0.58-1.18)	0.64 (0.35-1.18)	0.112
Multivariate adjusted OR^2^ (95% CI)	1 (Ref.)	1.07 (0.79-1.46)	0.84 (0.58-1.20)	0.66 (0.35-1.24)	0.161
Multivariate adjusted OR^3^ (95% CI)	1 (Ref.)	1.07 (0.79-1.46)	0.83 (0.58-1.20)	0.66 (0.35-1.23)	0.159
Multivariate adjusted OR^4^ (95% CI)	1 (Ref.)	1.08 (0.79-1.47)	0.84 (0.58-1.20)	0.66 (0.35-1.23)	0.200
Multivariate adjusted OR^5^ (95% CI)	1 (Ref.)	1.08 (0.79-1.47)	0.84 (0.58-1.20)	0.66 (0.35-1.24)	0.210

^a^One portion of white bread or whole-grain bread was specified as 60 g or 3 slices.

*OR* Odd Ratio.

*CI* Confidence Interval.

^1^adjusted by age, sex, physical activity, time spent in TV watching, total time of sedentary activities, smoking status, baseline BMI.

^2^adjusted by age, sex, physical activity, time spent in TV watching, total time of sedentary activities, smoking status, baseline BMI, fiber intake, and total energy intake.

^3^adjusted by age, sex, physical activity, time spent in TV watching, total time of sedentary activities, smoking status, baseline BMI, fiber intake, total energy intake, and protein percentage.

^4^adjusted by age, sex, physical activity, time spent in TV watching, total time of sedentary activities, smoking status, baseline BMI, fiber intake, total energy intake, and olive oil consumption.

^5^adjusted by age, sex, physical activity, time spent in TV watching, total time of sedentary activities, smoking status, baseline BMI, fiber intake, total energy intake, olive oil consumption, soft-drinks, and fast-food consumption.

When we adjusted for other potential confounding variables such as soft drinks and fast- food intake similar results were observed OR: 1.43; 95% CI: 1.11 to 1.86; P for trend = 0.015 (Table 4). Similarly, when we repeated the analyses including in the model percentage of energy from carbohydrates and from total fat the results were enhanced after adjusting for both macronutrients: adjusted OR: 1.73; 95% CI: 1.30 to 2.29, P for trend = 0.001.

We also adjusted for changes in physical activity after 2 years of follow-up and comparable results were obtained OR: 1.38; 95% CI: 1.06 to 1.79; P for trend = 0.029.

When we took into account duration of follow-up, we also obtained significant results: adjusted relative risk = 1.48; 95% CI: 1.13 to 1.92, P for trend = 0.008 (data not shown).

When we categorized participants according to quintiles of consumption of white bread, and we compared the highest quintile versus the lowest quintile, similar results were observed (OR: 1.33; 95% CI: 1.01 to 1.74) (data not shown).

A higher consumption of whole-grain bread was inversely associated with the risk of overweight/obesity although the association was not statistically significant.

When we excluded 572 postmenopausal women (n = 8695) similar results were observed both for white bread and for whole grain bread (OR: 1.31; 95% CI: 1.01 to 1.70, P for trend = 0.085 and OR: 0.58; 95% CI: 0.30 to 1.13, P for trend = 0.24, respectively) (data not shown).

Results did not change when we excluded participants with hypertension at baseline, when we stratified the sample by sex or when we excluded participants who had gain more than 3 kg in the last 5 years before entering the cohort (data not shown).

In this prospective cohort we have assessed the relationship between GI and GL and subsequent changes in body weight in a Mediterranean country and we have reported a significant association between white bread consumption and the incidence of overweight/obesity in a free-living population. In this considerably slim Mediterranean cohort of young adults completely composed of university graduates, a higher GI was not associated with a higher weight gain. On the contrary, GL was inversely associated with average yearly weight gain. In addition, the risk of overweight/obesity was neither associated with GL or GI.

To our knowledge, only two prospective studies have been conducted in a Mediterranean population, the EPIC cohort [11] and the PREDIMED trial [12]. Results from the EPIC study suggested that a low consumption of white bread may help to prevent abdominal fat accumulation among European men and women. The analysis in a subsample of participants of the PREDIMED trial, after 4 years of follow-up, reported that reducing white bread, but not whole-grain bread consumption, within a Mediterranean-style food pattern setting is associated with lower gains in weight and abdominal fat.

At the moment, although the potential benefits of a low GI and GL diets on weight gain have been hypothesized and that these diets can be useful for weight loss in obese subjects [25], epidemiological studies conducted in humans about this issue, most often with a cross-sectional design [26] have had inconsistent results to support a causal role of GL or GI on long-term body weight control among initially non-obese subjects [26, 27]. However, our findings are consistent with several cross-sectional studies and with a few longitudinal studies that have suggested that the GI may be not associated with body weight or weight changes [28]. Similarly, in a Mediterranean cross-sectional study [26] including 8 195 Spanish adults GL was negatively associated with BMI, after adjusting for total energy intake. GI was not associated with BMI in any model. In another cross-sectional study carried out in Italy [27] among 7 724 participants, GI and GL were inversely related to BMI and waist to hip ratio. Finally, a Greek investigation [29] suggested that carbohydrates had no positive association with obesity, in line with the results reported during the 90s by Nelson and Stubbs, although there are plausible mechanisms linking the development of certain chronic diseases with high-GI diets [2]. On the other hand, similarly, results of other previous cross-sectional studies on dietary GL and body weight change are consistent with our findings in adults. Thus, the study conducted by Du H, et al. found inverse associations between GL and weight change in the center of Florence [8]. The last cross-sectional study in British adults found independent positive associations of dietary GI and GL with general and central obesity [30]. Besides, in a recent study [31], a higher GL was associated with a healthy BMI.

There are two reasons that might explain our results. First, previous studies have suggested that in the context of a Mediterranean dietary pattern, such as the diet of our participants, rich in fruit, vegetable, cereals and legumes with high GL, the association between GI and GL and obesity may be null or inverse [25, 26, 29]. Thus, a high-GL diet may be a generally a more healthy diet, than a low-GL diet, because the possible effect of dietary GL alone on body weight change is less important than the overall dietary pattern or than individual nutrients or foods with higher GI or GL in this diet [28]. At the same time, the Mediterranean-type dietary pattern has been suggested as a healthy dietary pattern to prevent weight gain over time [32]. In addition, GL in a context of a Mediterranean dietary pattern was associated with fiber intake becoming from vegetables, fruits, and legumes. Fiber, as well as a better conformity with the overall Mediterranean dietary pattern, has been suggested to be a protective factor against weight gain. Second, the effect of high GL or GI diets on weight loss may be more marked in individuals with abdominal obesity than in individuals with very low baseline BMI, because in the first case they will likely have insulin resistant and a in consequence a higher GI/GL diet will have effect on weight control, while in the second case, the effect may be negligible [33]. However, when we analysed adjusting for protein percentage, results did not remain statistically significant. More studies in normal-weight subjects are needed to examine the relationship between GI or GL body weight and obesity development. In addition, in this same Mediterranean cohort of free-living participants, a high consumption of a single food item responsible for the main variability of the GI and GL, white bread, was significantly associated with overweight/obesity. Bread, especially whole-grain bread, was a fundamental food in the traditional Mediterranean diet and it was consumed in all meals. Although, in last decades bread consumption has decreased in Spain from 62 kg/person/year in 1987 to 52 in 2007 [34], the minimally processed whole grain products, typical of Mediterranean diet, are been replaced with refined grains. In the SUN cohort for example the consumption of white bread is significantly higher than the consumption of whole-grain bread (65 and 11 g/day respectively). Recent data of bread consumption in the general Spanish population showed that bread is the cereal with the highest consumption and the difference between white and whole-grain bread consumption was even higher: 77 and 6 g/day respectively [34]. This fact might have negative effects on several disease or conditions, including weight gain [33]. A potential mechanism to explain this association may be based on the extra calories ingested by participants with high consumption of white bread. It seems that to evaluate health effects of food rich in carbohydrates dietary GI or GL should never be used in isolation. Nature of carbohydrates, rather than the quantity, and the content of fiber and other micronutrients present in whole grain products, such as whole grain bread, are clearly important [33]. Several studies have suggested that, the change from white bread to whole-grain bread could reduce the risk of diabetes [35].

Strengths of this study included: its prospective design, the previous validation of the methods used to assess weight and physical activity, the large population-based size, the long follow-up period, and the control for an important number of potential confounders.

Also, there are some potential limitations in our study to take into account. First, we assessed associations between dietary GL or GI and obesity, through weight change, because other measures of adiposity were not available for the whole sample. Nevertheless, when we conducted additional analyses in a subsample of the participants included in the study with available information for waist circumference (n = 3,157) to assess central adiposity the results were very similar, although they did not achieve statistical significance. Second, the computation of the GI of the habitual diet was calculated by using only values from the GI tables of Atkinson, et al. [18] and not from Spanish tables. Third, we assessed dietary GL and GI using data from FFQs. Fourth, dietary assessment was conducted only at baseline. However, in the case that some participants may have changed their dietary habits, this misclassification is most likely expected to be non-differential and therefore would most probably underestimate the true relationship between bread consumption and overweight/obesity. Furthermore, we have conducted the analysis for white bread consumption and incidence of overweight/obesity restricting our follow-up only to the first two years, and the results were very similar: adjusted OR: 1.35; 95% CI: 1.09-1.67 for those who consumed ≥ 2 servings/day versus ≤ 1 serving/week. Therefore, even when the dietary assessment was closer to the incidence of overweight/obesity our results remained fairly robust and there is no need for an assumption on unchanged dietary habits in the long term.

Finally, it is not only the consumption of white bread but also the consumption of other foods with white bread that might increase the risk of overweight/obesity.

## Conclusions

Despite evidence that low-GI and/or low-GL diets are independently associated with a reduced risk of certain chronic diseases [2], our results suggest that dietary GI and dietary GL were not associated with increased weight gain or an increased risk of overweight/obesity development in a Mediterranean cohort of young adults with a low average BMI and with a high intake of fruits and vegetables. In contrast, a high consumption of white bread was a risk factor for overweight/obesity in the same population. However, further studies, in special intervention studies, are needed before including these measures in the dietary recommendations for healthy populations.

## Abbreviations

GI: Glycemic index
GL: Glycemic load
FFQ: Food-frequency questionnaire
SUN: Seguimiento Universidad de Navarra
MET: Metabolic equivalent score.
